# PReS-FINAL-2177: Safety and lack of autoantibody production following influenza H1N1 vaccination in patients with juvenile idiopathic arthritis (JIA)

**DOI:** 10.1186/1546-0096-11-S2-O12

**Published:** 2013-12-05

**Authors:** N Aikawa, C Goldenstein-Schainberg, M Vendramini, L Campos, C Saad, J Moraes, A Duarte, A Precioso, M Timenesky, E Bonfa, C Silva

**Affiliations:** 1 Reumatologia, Faculdade de Medicina, Universidade de Sao Paulo, Sao Paulo, Brazil; 2 Instituto Adolpho Lutz, Universidade de Sao Paulo, Sao Paulo, Brazil

## Introduction

Vaccination is an effective tool against several infectious agents including influenza. In 2010, the Advisory Committee on Immunization Practices (ACIP) recommended influenza A H1N1/2009 immunization for high risk groups, including juvenile idiopathic arthritis (JIA) patients and more recently the EULAR task force reinforced the importance of vaccination in immunosuppressed pediatric rheumatologic patients. We have recently shown that Influenza A H1N1/2009 vaccination generated protective antibody production with short-term safety profile among 93 JIA patients, but the possible impact of the vaccine in autoimmune response in JIA have not been studied. Therefore, we aimed to assess the production of some autoantibodies generated following influenza H1N1 vaccination in JIA patients.

## Objectives

To assess the autoimmune response and H1N1 serology following influenza H1N1 vaccination in patients with JIA.

## Methods

Cepa A/California/7/2009 (NYMC X-179A) anti-H1N1 was used to vaccinate JIA patients: 1 dose of immunization was given to all participants and those <9yrs of age received a second booster 3 weeks apart. Sera were analyzed before and 3 weeks following complete vaccination. Serology against H1N1 virus was performed by hemagglutination inhibition antibody assay, rheumatoid factor (RF) by latex fixation test, antinuclear antibodies (ANA) by IIF, IgM and IgG anticardiolipin (aCL) by ELISA.

## Results

Among 98 JIA patients that were vaccinated, 58 sera were available for this study. Mean age of 58 JIA patients was 23.9 ± 9.5 yrs, 38 were females and 20 males with mean disease duration of 14.7 ± 10.1 yrs. JIA subtypes were: 33 (57%) poliarticular, 10 (17%) oligoarticular, 6 (10%) systemic and 9 (16%) other. Sixteen patients were off drugs while 42 (72%) were under different pharmacotherapy: 32 (55%) were on 1 DMARD/IS, 10 (17%) on 2 DMARDs/IS, 19 (33%) antimalarials, 29 (50%) MTX, 8(14%) sulfasalazine, 6 (10%) anti-TNFs, 4 (7%) abatacept; no patient was using prednisone >0.5 mg/kg/d. Seroprotection rates against H1N1 influenza increased from 23 to 83% and seroconversion rates were achieved in 78% JIA. Prior to vaccination, 31(53.4%) JIA patients were ANA+, 6(10.3%) RF+, and 4 (7%) IgM + IgG aCL+. After complete H1N1 vaccination, positivity for ANA remained the same whereas 1 patient became negative for IgG aCL, and another for RF, IgM and IgG aCL. One (1.7%) patient turned low titer IgG aCL+.

## Conclusion

Vaccination of JIA patients against pandemic influenza A (H1N1) generated successful protective antibody production without the induction of autoantibody production, except for 1 patient that became positive for low titer IgG aCL, supporting its safety.

## Disclosure of interest

None declared.

